# Comparison of α-Tocopherol, α-Tocopherol Acetate, and α-Tocopheryl Polyethylene Glycol Succinate 1000 Absorption by Caco-2 TC7 Intestinal Cells

**DOI:** 10.3390/nu13010129

**Published:** 2020-12-31

**Authors:** Charlotte Cuerq, Claire Bordat, Charlotte Halimi, Emilie Blond, Marion Nowicki, Noël Peretti, Emmanuelle Reboul

**Affiliations:** 1 Biochemistry Department, Hospices Civils de Lyon, 69495 Pierre-Benite , France; charlotte.cuerq@chu-lyon.fr (C.C.); emilie.blond@chu-lyon.fr (E.B.); 2 CarMeN Laboratory, INSERM U1060, INRA UMR 1397, INSA-Lyon, Université Lyon 1, 60310 Pierre-Benite, France; claire.bordat@univ-amu.fr; 3 AMU, INRAE, INSERM, C2VN, 13005 Marseille, France; charlotte.halimi@univ-amu.fr (C.H.); marion.nowicki@univ-amu.fr (M.N.); 4 Pediatric Hepato-Gastroenterology and Nutrition Unit, Hôpital Femme Mère Enfant de Lyon, Hospices Civils de Lyon, 69677 Bron, France

**Keywords:** vitamin E, tocofersolan, TPGS, enterocyte, micelles, bioavailability, intestine

## Abstract

(1) Background: vitamin E is often supplemented in the form of tocopherol acetate, but it has poor bioavailability and can fail to correct blood tocopherol concentrations in some patients with severe cholestasis. In this context, α-tocopheryl polyethylene glycol succinate 1000 (TPGS) has been of value, but very little is known about the mechanisms of its absorption. The aim of our work was to evaluate the mechanisms of absorption/secretion of TPGS compared to tocopherol acetate (TAC) and α-tocopherol by human enterocyte-like Caco-2 TC7 cells. (2) Methods: two weeks post-confluence Caco-2 cells were incubated with tocopherol- or TAC- or TPGS-rich mixed micelles up to 24 h and, following lipid extraction, TAC and tocopherol amounts were measured by high performance liquid chromatography (HPLC) in apical, cellular, and basolateral compartments. (3) Results: at equivalent concentrations of tocopherol in the apical side, the amounts of tocopherol secreted at the basolateral pole of Caco-2 cells are (i) significantly greater when the tocopherol is in the free form in the micelles; (ii) intermediate when it is in the TAC form in the micelles (*p* < 0.001); and (iii) significantly lower with the TPGS form (*p* < 0.0001). Interestingly, our results show, for the first time, that Caco-2 cells secrete one or more esterified forms of the vitamin contained in TPGS at the basolateral side.

## 1. Introduction

**Figure 1:**
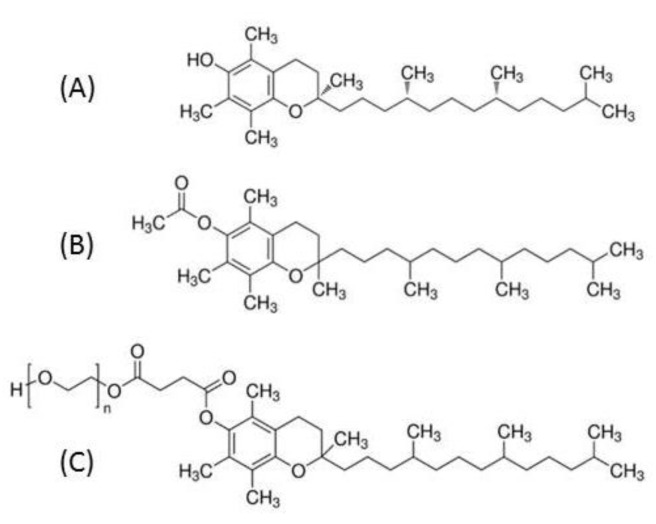
Schematic representation of tocopherol and its esterified derivatives; (**A**): α-Tocopherol, (**B**): α-Tocopherol acetate, (**C**): α-Tocopherol polyethylene glycol succinate 1000 (TPGS—*n* ≈ 23).

Vitamin E is a generic term for a group of eight fat-soluble compounds, the tocopherols and tocotrienols, of which α-tocopherol has the highest biological activity [1]. Alpha-tocopherol functions as an antioxidant that protects cell membranes from oxidative damage. Moreover, it plays a role in improving nerve conduction, and, in connection with vitamin A, is important for preserving normal vision [2]. During digestion, vitamin E is solubilized into mixed micelles in the presence of bile salts before being incorporated in chylomicrons in the enterocyte to be secreted into the circulation [3]. Normal vitamin E blood levels generally range from about 23 μmol/L to 46 μmol/L. The risk of vitamin E deficiency is of primary concern in malnutrition and can be caused by reduced dietary intake, inadequate bile-dependent intestinal absorption, insufficient pancreatic secretions, digestive resection or genetic diseases, such as ataxia with isolated vitamin E deficiency or intestinal genetic hypocholesterolemia (namely abetalipoproteinemia, hypobetalipoproteinemia, and chylomicron retention disease) [4]. In clinical nutrition, vitamin E is often supplemented in the form of tocopherol acetate (TAC) and the oral route is the preferred and often sufficient route of administration. Nevertheless, oral TAC has a poor bioavailability and can fail to prevent neurological complications in children with intestinal malabsorption of lipids, such as in severe cholestasis or intestinal genetic hypocholesterolemia [5]. In this context, α-tocopheryl polyethylene glycol succinate 1000 (TPGS, i.e., tocofersolan), a water-soluble derivative of RRR-α-tocopherol, has been developed and has shown its effectiveness in severe chronic cholestasis (Figure 1) [6]. Owing to its amphipathic structure, it forms micelles in the absence of bile acids allowing vitamin E to cross the unstirred water layer at the apical side of the intestinal mucosa and then be absorbed into the enterocyte. However, very little is known about the mechanisms of TPGS absorption. The aim of our work was to characterize the absorption of TPGS compared to TAC and α-tocopherol by the enterocytes, independently of the other digestive factors such as biliary micelle formation and pancreatic enzyme activity.

## 2. Materials and Methods

### 2.1. Chemicals

RRR-α-tocopherol (>99% pure) was a generous gift from DSM Nutritional Products Ltd. (Kaiseraugst, Switzerland). RRR-γ-tocopherol (>97% pure), DL-α-tocopherol acetate (>96% pure), 2-oleoyl-1-palmitoyl-sn-glycero-3-phosphocholine (phosphatidylcholine), 1-palmitoyl-sn-glycero-3-phosphocholine (lysophosphatidylcholine), monoolein, non-esterified cholesterol, oleic acid, sodium taurocholate, potassium hydroxide (KOH) and pyrogallol were from Sigma-Aldrich (Saint-Quentin Fallavier, France). TPGS was contained in VEDROP^®^ 50 mg/mL (Orphan Europe, Puteaux, France). Dulbecco’s modified Eagle’s medium (DMEM) containing 4.5 g/L glucose, trypsin-ethylenediaminetetraacetic acid (EDTA) (500 mg/L and 200 mg/L, respectively), penicillin/streptomycin, phosphate-buffered saline (PBS) were purchased from Life Technologies (Villebon-sur-Yvette, France). Fetal bovine serum (FBS) was from PAA (Vélizy-Villacoublay, France).

### 2.2. Preparation of Vitamin-Rich Micelles

For delivery of vitamin E forms to cells, molecules were incorporated into or dissolved with mixed micelles, which had a similar lipid composition to those found in vivo [7]. Micelles were prepared as previously described [8] to obtain the following final lipid concentrations: 0.04 mM phosphatidylcholine, 0.16 mM lysophosphatidylcholine, 0.3 mM monoolein, 0.1 mM non-esterified cholesterol, 0.5 mM oleic acid, and 5 mM taurocholate. Vitamin concentrations in the micellar solutions were checked by HPLC before each experiment.

### 2.3. Caco-2 TC7 Cell Culture

Cells were cultured in the presence of DMEM supplemented with 20% heat-inactivated FBS, 1% non-essential amino acid, and 1% antibiotics (complete medium), as previously described [8]. For each experiment, cells were seeded and grown on transwells as previously described to obtain confluent, differentiated cell monolayers [8]. Twelve h prior to each experiment, the medium used in apical and basolateral chambers was a serum-free complete medium. During preliminary tests, the integrity of the cell monolayers was checked by measuring trans-epithelial electrical resistance before and after the different experiments using a voltohmmeter fitted with a chopstick electrode (Millicell ERS; Millipore, Saint-Quentin-en-Yvelines, France).

### 2.4. Vitamin E Absorption by Caco-2 TC7 Cells

At the beginning of each experiment, cell monolayers were washed twice with 0.5 mL PBS. The apical side of the cell monolayers received either α-tocopherol (≈100 µM), TAC (≈100 µM), or TPGS (≈100 µM) micellar solutions at the apical side, whereas the other side received the serum-free complete medium. The concentration of 100 µM was chosen to have a good cellular amount of newly absorbed vitamin E as shown in our previous work [9] and to accurately measure basolateral efflux of vitamin E. Cells were incubated for up to 24 h at 37 °C. At time (t) = 0, 3 h, 6 h, 10 h, and 24 h, both apical and basolateral media were harvested. Cells were then carefully rinsed twice with 0.5 mL ice-cold PBS before being scrapped in 560 µL PBS. All samples were stored at –80 °C until further analysis.

### 2.5. Vitamin E Extraction and HPLC Analysis

- α-tocopherol and TAC

α-tocopherol and TAC were extracted from 25 µL to 1 mL aqueous samples using the following method. γ-tocopherol, which was used as an internal standard, was added to the samples in the same volume of ethanol. The mixture was extracted once with one volume of hexane. The hexane phase obtained after centrifugation (500× *g*, 10 min, 4 °C) was collected and evaporated to dryness under nitrogen. The dried extracts were dissolved in 200 µL methanol. A volume of 5–200 µL was used for HPLC analysis.

- TPGS

TPGS was assayed indirectly by quantifying α-tocopherol released after saponification by potassium hydroxide (KOH 5.5% + pyrogallol 1%) for 10 min at room temperature and by subtracting α-tocopherol determined before saponification.

### 2.6. Vitamin E HPLC Analysis

**Figure 2:**
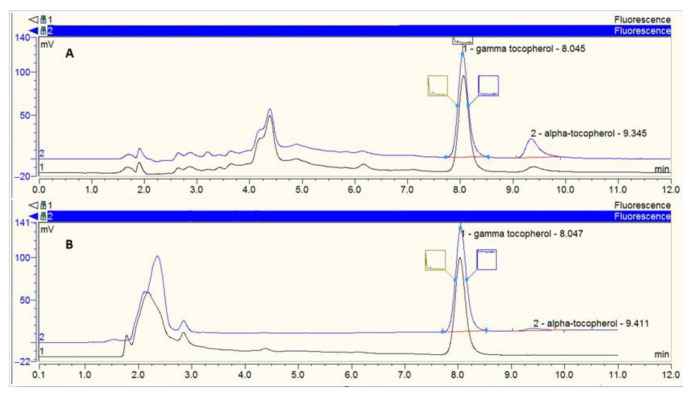
Example of chromatogram. (**A**) The upper part of the figure represents the chromatogram after analysis of the basolateral compartment under the condition with saponification (=hydrolysis of esterified tocopherol). The conditions at 3 h (h3) are shown in black and the conditions at 24 h (h24) are in blue on the same chromatogram. (**B**) The lower part of the figure represents the chromatogram after analysis of the basolateral compartment under the condition without saponification (=free tocopherol). The conditions at h3 are shown in black and the conditions at h24 are in blue on the same chromatogram. γ-tocopherol was used as an internal standard. The red lines represent the baselines of the chromatography peaks.

All the analyses were realized using a 250 × 4.6 nm RP C18, 5 µm Zorbax column (Agilent Technologies, Montpellier, France) maintained at a constant temperature (25 °C) and a guard column. Vitamin E analysis was performed with a 100% methanol mobile phase (flow rate = 1.5 mL/min). The HPLC system comprised a Dionex separation module (P680 HPLC Pump and ASI-100 Automated Sample Injector, Dionex, Aix-en-Provence, France), a Dionex UVD340U photodiode array detector (vitamin E detection at 292 nm) and a JASCO fluorimetric detector (JASCO, Nantes, France). For fluorimetric analysis, tocopherols were detected at 325 nm after light excitation at 292 nm. Vitamins were identified by retention time compared with pure standards. Quantification was performed using Chromeleon software (v6.50 SP4 Build 1000, Dionex) comparing peak area with standard reference curves (Figure 2).

### 2.7. Statistics

**Figure 3:**
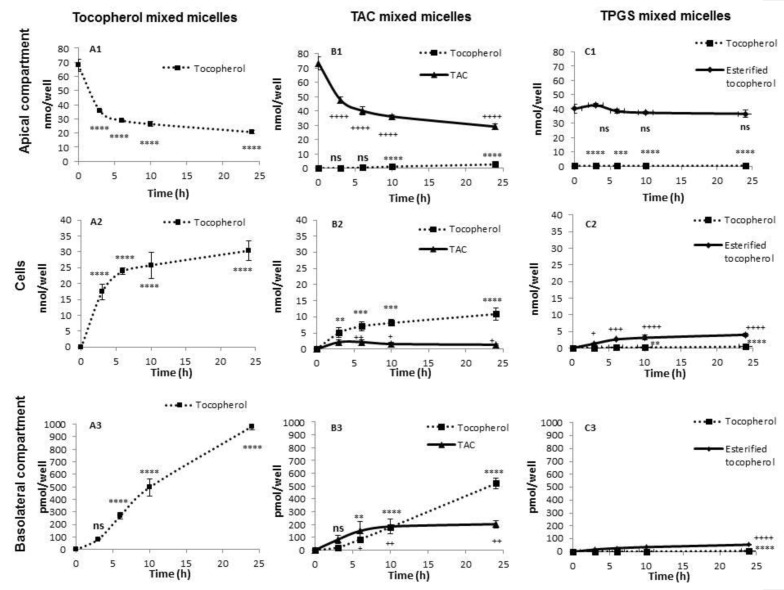
Uptake and secretion of different forms of tocopherol by Caco-2 cells. Two weeks post-confluence Caco-2 cells were incubated with tocopherol- or tocopherol acetate (TAC)- or α-tocopheryl polyethylene glycol succinate 1000 (TPGS)-rich mixed micelles up to 24 h and, following lipid extraction, TAC and tocopherol amounts were measured by HPLC in the different compartments. For the TPGS condition, esterified tocopherol was determined indirectly by measuring the tocopherol before and after saponification of the different compartments. (**A1**–**A3**) Tocopherol amounts in the apical, cellular, and basolateral compartment upon incubation with tocopherol-rich mixed micelles. (**B1**–**B3**) TAC and free tocopherol amounts in the apical, cellular, and basolateral compartment upon incubation with TAC-rich mixed micelles (**C1**–**C3**) Esterified tocopherol and free tocopherol amounts in the apical, cellular, and basolateral compartment upon incubation with TPGS-rich mixed micelles. For each vehicle and for each time point, *n* = 3. Values are means of amounts with their standard errors represented by vertical bars. Tukey test with time as a factor were carried out. ** *p* < 0.01; *** *p* < 0.001; **** *p* < 0.0001; ^+^ p < 0.05; ^++^ p < 0.01; ^+++^ p < 0.001; ^++++^ p < 0.0001 (* are for tocopherol and are replaced by ^+^ for TAC and esterified tocopherol); ns = non-significant. Abbreviations: TAC: tocopherol acetate; TPGS: α-tocopheryl polyethylene glycol succinate 1000.

**Figure 4:**
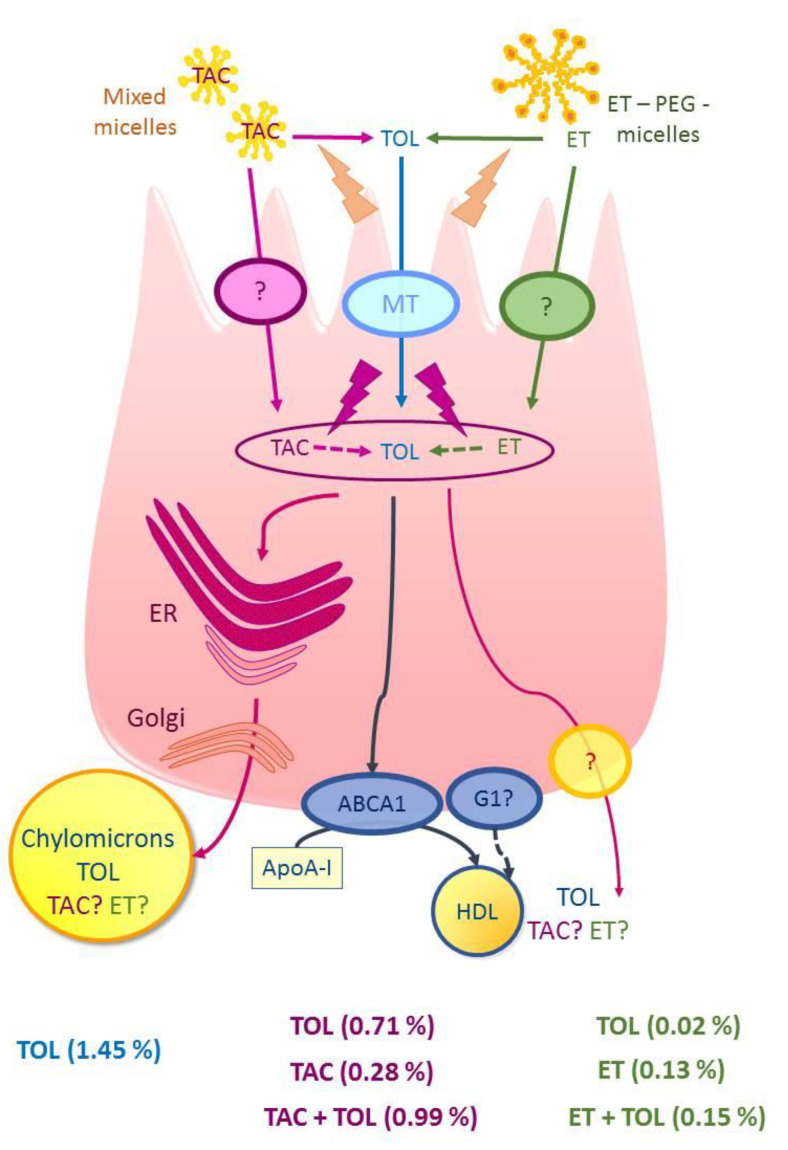
General view of the absorption/secretion of the different forms of tocopherol by the enterocyte. Consumption of α-tocopherol (TOL) leads to its uptake by the intestine and secretion in the circulation in chylomicron and HDL particles together with triacylglycerol and cholesterol. TAC and esterified tocopherol (ET) in the form of PEGylated α-tocopherol (ET-PEG, i.e.,TPGS) may be hydrolyzed to free α -tocopherol in the stomach by non-enzymatic hydrolysis, in the proximity of the brush border epithelium by esterase hydrolysis, and at the surface of the enterocytes via a lipase. Alternatively, entire TAC or ET can pass through cell membranes, thereby enabling the absorption of the intact TAC or ET molecule. A certain proportion of TAC and ET can then be secreted in their entire form. The percentages indicate the fraction secreted at h24 compared to the amount in the apical compartment at h0. TAC + TOL (or ET + TOL) indicates the tocopherol equivalent from the apical amount of TAC (or ET-PEG). Abbreviations: ABCA1: ATP-binding cassette A1; ApoA-1: apolipoproteine A1; ER: endoplasmic reticulum, ET: esterified tocopherol; G1: ATP-binding cassette G1; HDL high density cholesterol; MT: membrane transporter; PEG: polyethylene-glycol; TAC: tocopherol acetate; TOL: α-tocopherol; TPGS: α-tocopheryl polyethylene glycol succinate 1000.

Results are expressed as means of amounts with their standard errors (Figure 3). The percentage of vitamers secreted at the basolateral compartment at h24 is the ratio of the amounts of vitamers between the basolateral compartment at h24 and the apical compartment at h0. Under the TAC and TPGS conditions, the percentages were calculated in two forms: (i) the percentage of TAC secreted in its entire form and (ii) the percentage of tocopherol equivalent, which represents the concentrations of TAC (or esterified tocopherol) in its entire form as well as free tocopherol released by hydrolysis (Figure 4).

Differences in vitamer apical decrease, cellular uptake, and basolateral efflux were tested using analysis of variance (ANOVA—fixed-effects models). Prior to ANOVA, data were tested for equality of variances. Tukey’s test was used as a post hoc test for pairwise comparisons. Statistical analyses were performed using GraphPad Prism 9.0 (GraphPad Software, LLC; San Diego, CA, USA). Values of *p* < 0.05 were considered significant.

## 3. Results

### 3.1. α-Tocopherol Absorption by Caco-2 TC7 Monolayers

In the control condition with micelles containing α-tocopherol, a decrease in the tocopherol amount over time was observed at the apical site of the Caco-2 cells (from 68 nmol/well at h0 to 21 nmol/well at h24; *p* < 0.0001) (Figure 3(A1)). This decrease was concomitant with the appearance of tocopherol up to 30 nmol/well in Caco-2 cells (*p* < 0.0001) (Figure 3(A2)) and up to 983 pmol/well at the basolateral site (*p* < 0.0001) (Figure 3(A3)).

### 3.2. TAC

Concerning the condition using mixed micelles enriched with TAC, a decrease in TAC amount concomitant with the appearance of free α-tocopherol at the apical side was observed (from 73 and 0 nmol/well at h0 to 29 and 2.6 nmol/well at h24 of TAC and free α-tocopherol, respectively; *p* < 0.0001) (Figure 3(B1)). At the cellular level, an increase in the concentrations of TAC and free tocopherol was observed over time (*p* < 0.0001) (Figure 3(B2)). Finally, at the basolateral side, tocopherol was secreted predominantly in the form of free α-tocopherol (520 pmol/well at h24; *p* < 0.0001) (Figure 3(B3)). Interestingly a small amount of TAC was found at the basolateral level tocopherol (202 pmol/well at h24; *p* < 0.0001) (Figure 3(B3)).

### 3.3. TPGS

Finally, when TPGS was diluted into mixed micelles, the amounts of TPGS and free α-tocopherol hardly varied over time at the apical side (from 40 to 36 nmol/well at h0 and h24, respectively, for TPGS; *p* < 0.0001) (Figure 3(C1)). The vitamin E content in the cells (Figure 3(C2)) as well as on the basolateral side (Figure 3(C3)) was predominantly in the form of esterified tocopherol (4 nmol/well and 54 pmol/well at h24 in cellular and basolateral compartment respectively; *p* < 0.0001).

### 3.4. Percentage of Secretion of Each Vitamers

At equivalent amounts of tocopherol at the apical side, the amounts of tocopherol secreted at the basolateral pole of Caco-2 cells are (i) significantly greater when the tocopherol is in the free form in the micelles (% tocopherol secreted = 1.45%); (ii) intermediate when it is in the TAC form in the micelles (% tocopherol + TAC secreted = 0.99%) (*p* < 0.001); and (iii) significantly lower with the TPGS form (% tocopherol + esterified secreted = 0.15%) (*p* < 0.0001) (Figure 4).

## 4. Discussion

The objective of this work was to compare the absorption/secretion of RRR-α-tocopherol, dl-α-tocopherol acetate and dl-α-tocopheryl polyethylene glycol 1000 succinate by Caco-2 cells. It is often suggested that tocopherol esters should be hydrolyzed prior absorption by the intestine [3]. However, this hydrolysis by digestive enzymes may be incomplete and a fraction of the ingested tocopherol esters may reach the brush border membrane of the enterocytes.

The fact that two molecules used were in dl form and the other in RRR form has no impact on the results insofar as the intestinal cells do not discriminate against the various isomers of vitamin E [10,11]. We incorporated or diluted the different vitamers in mixed micelles, as such vehicles containing bile salts and fatty acids are (i) present during the postprandial period, and (ii) required for both correct uptake and secretion in chylomicron-like structures by Caco-2 cells [12,13]. Caco-2 cell secretion to the basolateral compartment is usually low, but allows comparison between different conditions [9,12].

Our main results are: (1) the absorption of TPGS and TAC are efficient, and partly requires a hydrolysis at the apical pole of Caco-2 cells; (2) TAC absorption into the enterocyte partially occurs in its entire form; (3) TPGS absorption and secretion are much lower than those of TAC and α-tocopherol; (4) and vitamin E can be secreted at the basolateral side in esterified forms (Figure 3 and Figure 4).

The Caco-2 cell is a model that enables the study of the enterocyte phase of digestion. In vivo, both TPGS and TAC are submitted to digestive enzymes (salivary, gastric, and pancreatic lipases) and bile salts. The supplied form of α-tocopherol, either as a free molecule or as TAC is of particular importance for its bioavailability. In animals, when vitamin E is ingested alone, the net uptake of alpha-tocopherol from the free phenol form is only half that from the acetate form [14]. However, in animals and in humans, when given with a meal, the competitive uptake studies of the two forms showed that the absorption of the 2 forms (free phenol or TAC) was equivalent [14]. This difference in our results highlights the importance of the intraluminal steps for vitamin E absorption.

Our results concerning the absorption of α-tocopherol and TAC are consistent with the prior literature [8,9,15]. The fact that TAC is hydrolyzed at the apical pole of Caco-2 cells and partially penetrates in its entire form into these cells has already been reported [15,16]. This hydrolysis is compatible with the presence of an esterase at the brush border or in the cytosol of Caco-2 cells. Several enzymes are candidates, including cholesterol ester hydrolase or carboxyl esterase 1 [15,16,17,18,19]. Furthermore, studies demonstrated the presence of a hydrolase activity directed against retinol esters at the brush border of rat and human enterocytes [20,21,22]. The secretion at the basolateral pole of TAC in its intact form has been reported by Brisson et al. in quantities greater than those found in our work (0.3 nmol of vitamin E/cm2 of cells versus 0.2 nmol/well) [15]. However, recent work by Desmarchelier et al. did not allow detection of TAC at the basolateral pole of Caco-2 cells with micelles containing 20 µmol/L of TAC, an incubation time of 6 h and a detection limit of 62.5 pmol/well [16]. Despite these discrepancies, our results are consistent with these observations since (i) the micelles used in our work contained nearly four times more TAC, and (ii) the incubation times were longer, likely inducing a greater absorption of vitamin E. In addition, at 6 h, we found 81 pmol/well on average of TAC, i.e., a quantity very close to the detection limit set by Desmarchelier et al. [16].

Interestingly, our results show, for the first time, that Caco-2 cells secrete one or more esterified forms of the vitamin contained in TPGS at the basolateral side. Traber et al. previously reported that TPGS could be taken up in its entire form by Caco-2 cells but did not evaluate its secretion at the basolateral side [23]. They demonstrated that increasing concentrations of TPGS increased proportionally the total cellular tocopherol content in cultured fibroblasts. When the fibroblasts were incubated in the presence of TPGS for 4 h, the tocopherol contained in the cells was almost entirely in the form of esterified tocopherol. As the incubation time increased to 24 h, the amount of tocopherol in free form increased but remained much less than the amount of esterified tocopherol. This phenomenon was also dependent on the temperature. In order to be able to characterize whether the esterified tocopherol found in the fibroblasts was in the form of tocopherol succinate or TPGS, the authors synthesized 14-carbon-labeled TPGS (14C-TPGS) from unlabeled tocopherol succinate and labeled polyethylene-glycol 1000 (PEG1000—14C-PEG). The amount of esterified tocopherol present in the Caco-2 cells was equivalent to the amount of 14C, unequivocally indicating that the TPGS had been taken up by the Caco-2 cells in its entire form. In our work, micelles were made with TPGS from the drug VEDROP^®^ and we cannot affirm that the vitamin E quantified after saponification comes from the TPGS only. The results therefore indicate a concentration of esterified tocopherol that may correspond to either mono- or di-TPGS as well as to tocopherol succinate, even if the concentrations of these last two molecules are in the minority. Our results suggesting that TPGS is partially absorbed in its intact form are also consistent with the work of Folmer and colleagues [19]. By synthesizing tocopherol esters with PEG chains containing 6 or 12 ethylene oxide units, the authors demonstrated that more than half of the tocopherol contained in Caco-2 cells after 24 h of incubation with mixed micelles was found in its intact esterified form. The esterified molecule of smaller size was found in larger quantity. The other half was a mixture of tocopherol and tocopherol succinate. According to their modeling work, it would appear that tocopherol derivatives displaying less than eight ethylene oxide units are preferentially hydrolyzed to tocopherol, while longer compounds are preferentially hydrolyzed to tocopherol succinate. In their model with micelles containing the synthesized molecules at a concentration of 26 µmol/L, no molecule was detected at the basolateral side, either in free or esterified form. This may be due to the fact that the concentration used was lower than in our study. Furthermore, it seems unlikely that the presence of esterified tocopherol at the basolateral side is due to cell damage insofar as cytotoxicity tests based on the release of lactate deshydrogenase (LDH) by Caco-2 cells have shown that TPGS 1000 is cytotoxic at concentrations much higher (625 µmol/L) than those used in our experiments (100 µmol/L) [24].

Finally, Sokol, in his work on the use of TPGS in the treatment of children with cholestasis, performed loading doses of TPGS at a dose of 100 IU/kg (maximum 2000 IU) in adult controls and children with cholestasis [25]. He showed that a very small fraction of PEG 1000 was found in the urine (1.7 and 3% of the ingested amount in children with cholestasis and in control adults, respectively). The authors considered that the PEG 1000 found in the urine was derived from the PEG absorbed following the hydrolysis of the TPGS before it entered the enterocyte. In fact, prior work had shown that after oral loading with 10 g of PEG 1000: 37% and 48.6% of PEG 1000 was found in the urine of patients with Crohn’s disease and of controls respectively [26]. The PEG 1000 found in the urine by Sokol following the administration of TPGS may actually come from the hydrolysis of the TPGS following its secretion in entire form by the enterocytes, as shown in our study on a Caco-2 cell model.

Overall, our work shows that at equivalent concentrations of tocopherol at the apical side, the amounts of tocopherol secreted at the basolateral pole of Caco-2 cells are (i) significantly greater when the tocopherol is the in free form in the micelles; (ii) intermediate when it is in the TAC form in the micelles (*p* < 0.001); and (iii) significantly lower with the TPGS form (*p* < 0.0001) (Figure 4). These results are consistent with the literature reporting that supplementation with tocofersolan had minimal effect on plasma α-tocopherol in adults with normal intestinal absorption, whereas similar doses of TAC resulted in a significant increase in plasma α–tocopherol [27]. These data are also consistent with our recently published work that showed that TAC was better absorbed than tocofersolan after a single oral dose in healthy volunteers [28]. One hypothesis to explain the lower absorption of TPGS compared to the TAC could be the steric hindrance of the PEG chain, which would limit (i) its absorption at the enterocyte level, and (ii) its access to an esterase to release tocopherol. In the future, studies with patients are needed to evaluate the differences of intestinal absorption of these molecules and the long-term consequences in growth, and neurological, visual, and hepatic evolution.

## Data Availability

The data presented in this study are available on request from the corresponding authors.
